# Oncological outcome of vocal cord-only radiotherapy for cT1-T2 glottic laryngeal squamous cell carcinoma

**DOI:** 10.1007/s00405-023-07904-2

**Published:** 2023-03-07

**Authors:** Mischa de Ridder, Johannes A. Rijken, Hilde J. G. Smits, Ernst J. Smid, Patricia A. H. Doornaert, Remco de Bree

**Affiliations:** 1 Department of Radiotherapy, University Medical Center Utrecht, Heidelberglaan 100, Postbox 85500, 3508 GA Utrecht, The Netherlands; 2 Department of Head and Neck Surgical Oncology, University Medical Center Utrecht, Utrecht, The Netherlands

**Keywords:** Radiotherapy, Glottic cancer, Larynx, Vocal cord, VMAT

## Abstract

**Purpose:**

Early-stage glottic cancer can be treated with radiotherapy only. Modern radiotherapy solutions allow for individualized dose distributions, hypofractionation and sparing of organs at risk. The target volume used to be the entire voice box. This series describe the oncological outcome and toxicity of individualized vocal cord-only hypofractionated radiotherapy for early stage (cT1a-T2 N0).

**Methods:**

Retrospective cohort study with patients treated in a single center between 2014 and 2020.

**Results:**

A total of 93 patients were included. Local control rate was 100% for cT1a, 97% for cT1b and 77% for cT2. Risk factor for local recurrence was smoking during radiotherapy. Laryngectomy-free survival was 90% at 5 years. Grade III or higher late toxicity was 3.7%.

**Conclusion:**

Vocal cord-only hypofractionated radiotherapy appears to be oncologically safe in early-stage glottic cancer. Modern, image-guided radiotherapy led to comparable results as historical series with very limited late toxicity.

## Introduction

Early-stage glottic laryngeal squamous cell carcinoma represents about 80% of all laryngeal carcinomas [1]. Most patients present with early-stage disease (stage I–II), because glottic tumors cause dysphonia even with a small irregularity of the vocal cord. Main risk factors for laryngeal cancer are smoking [2] and/or alcohol consumption [3, 4]. Predominantly, males are diagnosed with laryngeal cancer [5]. In the Netherlands, there is a trend towards decreased incidence, mainly in male patients [6], presumably because of a stricter smoking policy in public areas, and further discouragement from the government since last decades. Treatment of early-stage laryngeal cancer is either surgically or with radiotherapy. In choosing one treatment over another, one should consider the cure rate, larynx preservation rate, post-treatment voice quality, morbidity and treatment costs. Surgery, by means of transoral microscopic laser surgery (MLS), is mostly the preferred treatment option for medically fit patients, with cT1a (limited to one cord) carcinomas. Although radiotherapy results in similar oncological outcomes [7], for most patients the one-stop benefit of MLS outweighs radiotherapy in most of the cases. In addition, there are some hints that MLS for cT1a glottic cancer may have a slightly higher larynx preservation rate compared to radiotherapy [8], and total costs are lower for MLS than for radiotherapy [9]. Furthermore, transoral microscopic laser surgery can be repeated in selected cases. For some patients, surgery under general anesthesia may not be feasible due to severe comorbidity. In addition, surgery is not always technically feasible due to insufficient exposure, especially due to limited view of the anterior commissure caused by patient-related factors, e.g. limited mobility of the neck, retrognathia, a smacking jaw, a large tongue, or combinations of these. Peroperatively, if there is insufficient exposure, it is decided not to perform laser surgery, but only to take a biopsy to confirm the diagnosis of cancer and to register the patient for treatment by means of radiotherapy. If a tumor spreads via the anterior commissure to the contralateral side, a two-temp MLS may be considered to avoid webbing at the anterior commissure site, resulting in a poorer voice, making radiotherapy treatment a good alternative.

For patients with cT1b or cT2, clear data about larynx preservation and primary surgery is lacking [10, 11] and most institutes will advise radiotherapy in such cases. Especially for cT2 tumors, or tumors located in the anterior commissure it might be challenging to achieve adequate surgical margins with acceptable remaining glottic function. Long-term functional outcome after radiotherapy for cT1b or cT2 is good [12]. Individual patient or physician preferences could be of influence on this decision, so well-informed shared decision making plays an important role in regional treatment variation of cT1b and cT2 glottic cancer patients.

Historically patients were irradiated with opposing lateral fields covering the entire voice box. With the introduction of 3D-conformal and intensity modulated radiotherapy (IMRT), dose distributions improved and organ-at-risk (OAR) dose decreased [13]. With this improvement in dose distributions, hypofractionated radiotherapy was possible and found to be equally effective or maybe even better in terms of oncological outcome [14]. Improved diagnostic imaging and the possibility of image-guided radiotherapy (IGRT) opened up the way to rethink target definition for early-stage glottic laryngeal squamous cell carcinoma. For T1a carcinomas, the group from Erasmus University in Rotterdam (the Netherlands) developed a novel technique called single vocal cord irradiation (SVCI) [15]. With this technique, only the affected vocal cord is irradiated with limited margins. Local control, voice outcome and toxicity of this technique appeared to be excellent [15]. Studies that correlate histopathology with imaging (computed tomography (CT) and magnetic resonance (MR) imaging) showed that it is not necessary to include to entire voice box as clinical target volume (CTV), but a 6 mm margin is appropriate to cover all microscopic spread [16, 17]. In our institute, we started with hypofractionated vocal cord-only irradiation for early-stage glottic cancer in 2014.

The aim of this study was to evaluate the oncological outcome and toxicity of vocal cord-only hypofractionated radiotherapy for cT1a-T2 glottic laryngeal squamous cell carcinoma.

## Patients and methods

### Patients

Between 2014 and 2020, 93 patients with cT1a-T2 N0 glottic laryngeal squamous cell carcinoma were treated with vocal cord-only radiotherapy in our department. All patients were previously unirradiated. Previous laser surgery for another vocal cord lesion was not an exclusion criterion, but there had to be (a new) macroscopic tumor. Patients that needed postoperative radiotherapy after laser surgery were not included. All patients had histopathological proof of squamous cell carcinoma. Patients with carcinoma in situ (severe dysplasia) were excluded.

Permission for retrospective data collection was given by the Medical Ethical Committee of the Utrecht University Medical Center.

### Diagnostic workup, treatment and follow-up

All patients underwent full examination by a head and neck surgeon and radiation oncologist with flexible laryngoscopy and when in doubt examination under general anesthesia. Since 2020 we have started using office-based narrowband imaging (NBI) assessment of the vocal cords. Imaging of the larynx was performed using CT scans and in limited cases with MRI. Only for cT2-tumors, cervical lymph node examination was done with ultrasound.

Patients with a cT1b, low volume (< 2 cc) cT2 with normal vocal cord mobility or cT1a not suitable for laser excision due to tumor volume, exposure, anatomy or comorbidity were offered vocal cord-only radiotherapy. All patients were immobilized using a thermoplastic 5-point mask with slight hyperextension of the neck and individualized base. All imaging was performed in treatment position.

**Fig. 1:**
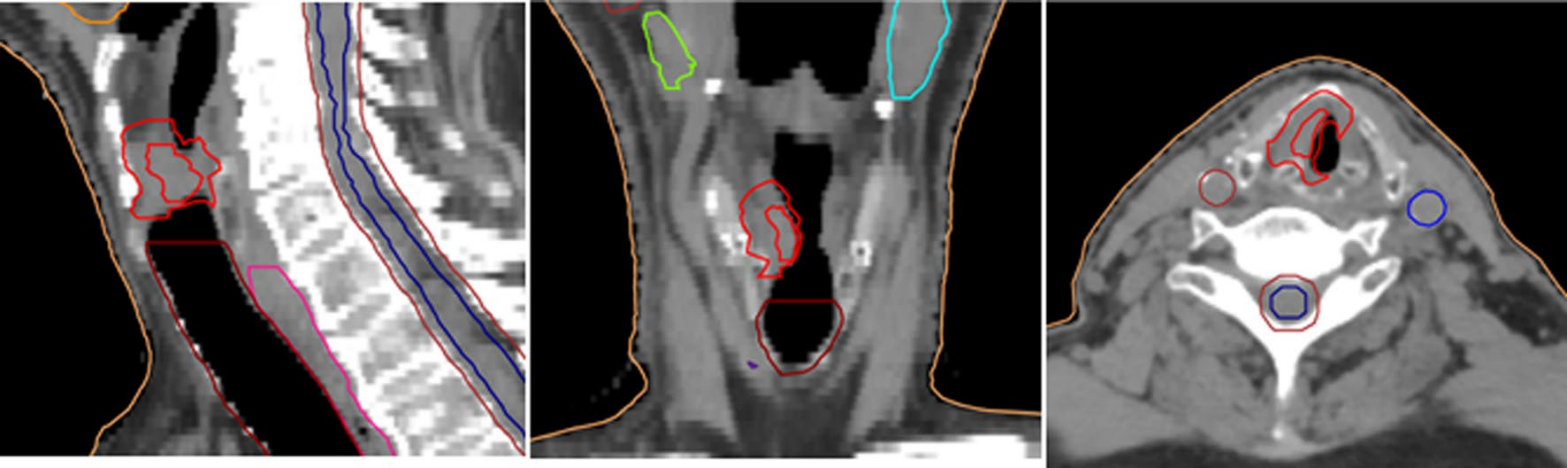
An example of the target volume definition of an early glottic cancer. The inner red line represents the GTV, whereas the outer red line represents the CTV. Other delineations are OARs

Gross tumor volume (GTV) was defined based on clinical examination as well as imaging. The clinical target volume (CTV) margin was 5 mm with a breathing motion 4D-CT-based internal target volume (ITV) (until mid-2018) or 6 mm isotropic margin (from mid-2018 till 2020). The planning target volume (PTV) was 3 mm left–right, 4 mm anterior–posterior and 6–8 mm cranio-caudal. The CTV was cropped within the laryngeal cartilage and excluding air cavity (Fig. 1).

Prescribed total dose was 60 Gy in 25 fractions of 2.4 Gy, which is an EQD2 (a/b 10 Gy) of 62 Gy and a BED (a/b 10 Gy) of 72 Gy.

Treatment planning was done using a 2 arc volumetric arc therapy (VMAT) technique with 6 MV photons, aiming at a coverage of the V95% of the PTV of 98%.

Setup verification was done by online correction protocol with cone beam CT before each fraction.

Patients underwent 5 fractions each week with an overall treatment time of 33–35 days.

Follow-up was standardized for 5 years with alternating visits at the departments of head and neck surgical oncology and radiotherapy. In the first year, patients were checked every 2 months, in the second year, every 3 months, in the third year, every 4 months and the last 2 years, every 6 months. Each consecutive follow-up visit consisted of physical examination with flexible laryngoscopy.

### Study endpoints

Primary endpoint was local control.

Secondary endpoints were disease specific and overall survival, grade III late toxicity and regional control.

The following grade III (CTCAE 4.0) toxicities were retrospectively scored: soft tissue ulcer, hypothyroidism, dysphagia and radionecrosis.

### Statistical analysis

Follow-up time was calculated from end of radiotherapy till date of last follow-up visit, death, tumor progression, or diagnosis of a new primary tumor. For overall survival, laryngectomy-free survival and local control, the Kaplan–Meier method was used to estimate the actuarial rates. *p*-values < 0.05 were considered as statistically significant. Toxicity was evaluated upon last follow-up visit, death, tumor progression, or diagnosis of a new primary tumor, whichever came first. Baseline risk factors for developing local recurrences were evaluated using Cox regression. For the statistical analyses, SPSS software was used (version 25, IBM Corporation, Armonk, NY).

## Results

A total of 93 patients were included in the study. The median follow-up of patients was 48 months with a range of 2–94 months. A total of 6 patients were lost to follow-up.

Baseline characteristics of the patients are shown in Table 1. The majority (84%) had been active or prior smokers at baseline and 17 patients (18%) continued smoking during radiotherapy.

**Table 1:** Patient and treatment characteristics

Gender		
Male	84	90%
Female	9	10%
Age		
Median	68	
Range	48–91	
cT		
cT1a	30	32%
cT1b	33	36%
cT2	30	32%
ACI		
Yes	29	31%
No	64	69%
Prior laser		
Yes	19	20%
No	74	80%
Smoking		
Active	31	33%
Prior	47	51%
No	15	16%
Smoking during RT		
Yes	17	18%
No	76	82%
GTV volume		
Mean	1.88	
Range	0.34–7.10	
CTV volume		
Mean	15.09	
Range	2.58–38.65	
PTV volume		
Mean	31.72	
Range	12.79–63.88	

*ACI* anterior commissure involvement, *RT* radiotherapy, *GTV* gross tumor volume, *CTV* clinical target volume, *PTV* planning target volume

Clinical T-stages were 30 cT1a (32%), 33 cT1b (36%) and 30 cT2 (32%). About a third (*n* = 29) of the patients had tumor involvement of the anterior commissure (ACI).

The mean GTV volume was 1.88 cc and the mean PTV volume was 31.7 cc. In total, 33 patients (36%) had a tumor volume above 2 cc. All patients finished the course of radiotherapy with a median overall treatment time of 33 days (range 32–37).

### Local control

In total, 7 patients developed a local recurrence. Of these, 5 were isolated local recurrences, 1 patient had local and distant failure, and 1 patient had local, regional and distant recurrent disease. All local failures were within the first 2 years. Local control at 5 years was 92%.

**Fig. 2:**
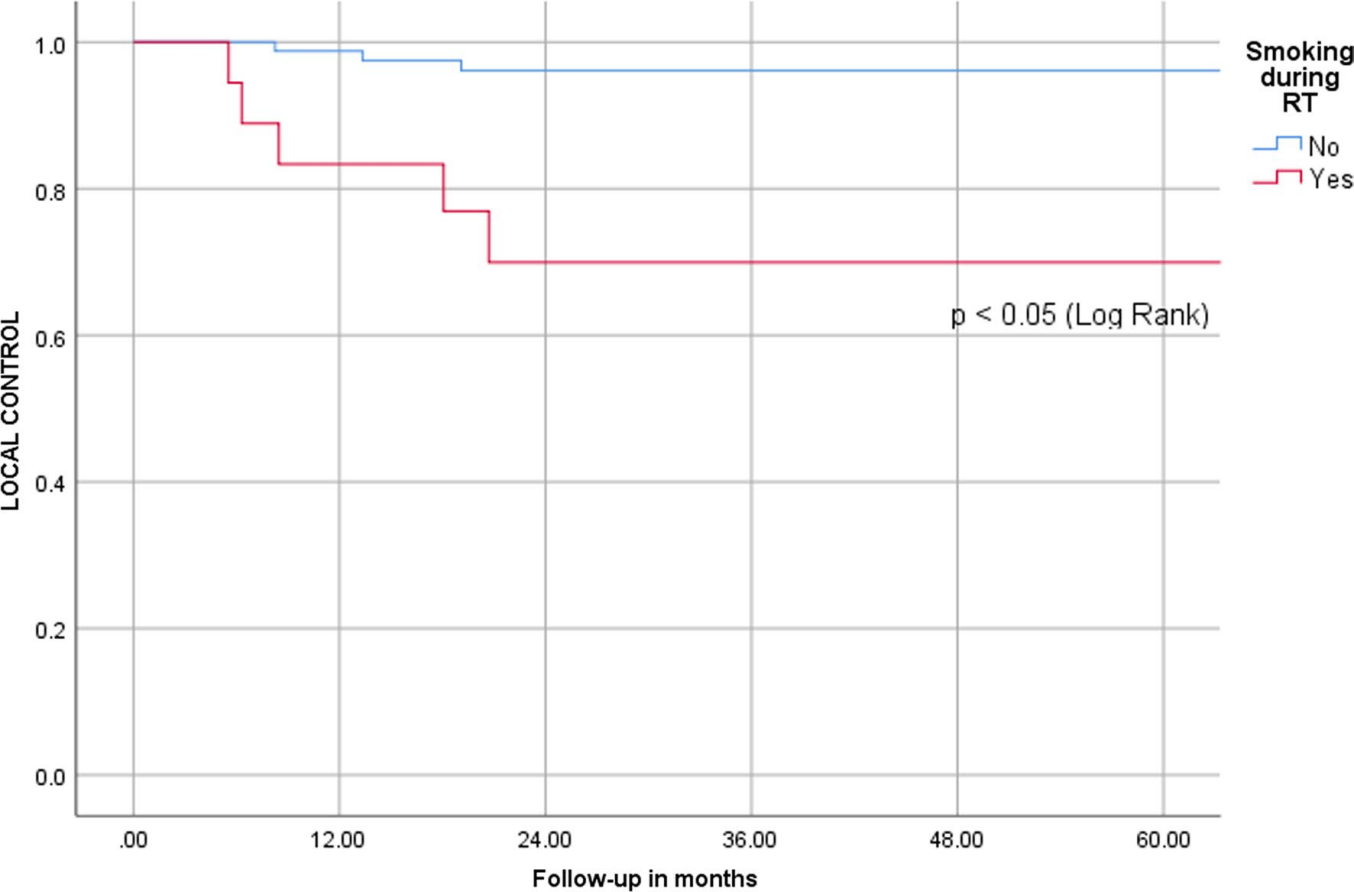
A Kaplan–Meier plot estimating local control for patients that smoke during radiotherapy compared to patients that did not smoke during radiotherapy

Patients with cT1a disease had a local control at 5 years of 100%, for cT1b disease, it was 97% and for cT2, it was 77% (Fig. 2).

All 5 patients with isolated local failure underwent salvage total laryngectomy and were alive at last follow-up (median 49 months). The 5-year local control after irradiation and including patients with salvage surgery was 98%.

One patient with local failure underwent salvage laryngectomy and is still alive after treatment with pembrolizumab (FU 21 months) for subsequent distant metastases. Another patient with local, regional and distant failure died of disease.

**Fig. 3:**
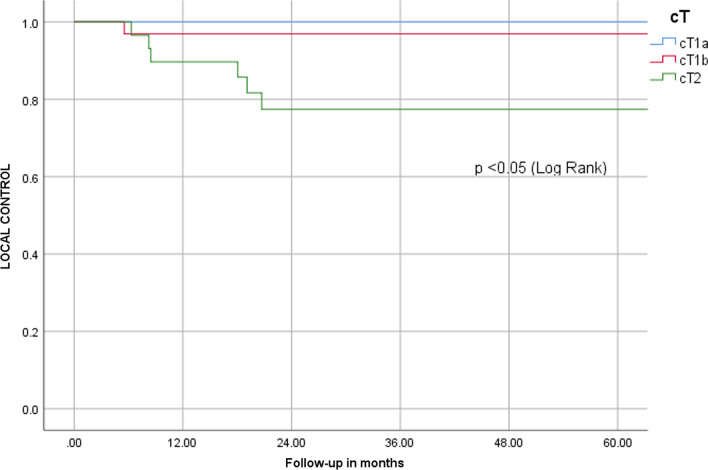
A Kaplan–Meier plot for local control by T-stage

Univariable Cox regression analyses showed the following risk factors for local recurrence (Table 2): cT2 stage (HR 8.0, 95% CI 1.6–39.8) and smoking during radiotherapy (HR 9.2, 95% CI 2.2–38.3) (Fig. 3). Patients with a tumor volume below 2 cc showed a non-significant trend for superior local control (95% vs. 85%, *p* = 0.194). Small numbers did not allow any further subgroup analyses.

**Table 2:** Univariate Cox’s proportional hazard model for local recurrence

	HR (95% CI)	*P*-value
Age	0.98 (0.91–1.06)	0.629
Gender		
Male (ref)		
Female	0.99 (0.12–8.0)	0.992
Stage		
cT1a/cT1b/cTis (ref)		
cT2	8.0 (1.6–39.8)	0.011
ACI		
Not involved (ref)		
Involved	0.7 (0.2–2.9)	0.623
PTV volume	1.02 (0.97–1.07)	0.385
Smoking during RT		
No smoking (ref)		
Continued smoking	9.2 (2.2–38.3)	< 0.001

*ACI* anterior commissure involvement, *ref* reference

### Regional control

In total, 4 patients developed a regional recurrence. In 1 patient, this was an isolated neck recurrence, for which a salvage neck dissection was performed 21 months after initial treatment and the patient remained free of disease (FU after neck dissection 33 months). Three patients with regional and distant recurrence were treated with reirradiation, but died of disease.

The actuarial regional control rate at 5 years of the entire cohort was 95%.

There was no difference between initial T-stage groups and risk of nodal recurrence.

### Toxicity

Total number of patients with grade 3 or higher late toxicity was 4 out of 93 (4.3%). One patient (1.1%) developed laryngeal cartilage necrosis 6 months after radiotherapy, for which the patient was treated with hyperbaric oxygen therapy and laryngectomy. The pathology report of the laryngectomy showed no vital tumor, only radiation induced necrosis.

In total, 3 patients (3.2%) developed late laryngeal edema (> 2 years after treatment) for which a temporary tracheostomy was needed. All patients were decannulated and free of tracheostomy at last follow-up. Two of these patients were also in need for temporary tube feeding during the period of edema. None of the patients developed hypothyroidism during follow-up.

### Survival

Of the 93 patients, a total of 25 patients died during follow-up. The estimated 5-year overall survival was 64%. Overall survival was not impacted by T-stage (*p* = 0.974).

Of the 27 patients who died during follow-up, 3 patients died of their larynx tumor and 24 of intercurrent disease. Estimated 5-year disease-specific survival was 96%.

Laryngectomy-free survival was 90% at 5 years.

## Discussion

This series describes the oncological outcome of 93 patients with early-stage glottic laryngeal squamous cell carcinoma treated with vocal cord-only radiotherapy (treated between 2014 and 2022 in our department). It shows excellent oncological outcomes with an estimated disease specific survival of 96% at 5 years. Patients with cT1a and cT1b disease had a local control at 5 years of 100% and 97%, respectively. For patients with cT2 tumors, this was 77%. These oncological outcomes are comparable with historical series published [18–23]. A study from Chera et al. included 585 patients treated in a single institution between 1964 and 2006 [20]. All of the patients were treated with radiotherapy only, covering the entire larynx. The local control rates for T1a (95%) T1b (94%), T2 (74–81%) and the results of our study are comparable. A more recent study from Elicin et al. included a total of 761 patients treated between 1990 and 2015 [22]. Patients were included from 10 different centers across Europe. In this study, there was some heterogeneity in fractionation and dose, but all patients were treated with whole larynx radiotherapy. Reported local control was comparable, possibly slightly lower (cT1a 89%, cT1b 83% and cT2 79%) compared to the present study. An Italian bi-institutional series of 256 patients with cT2 glottic cancer showed a local control rate of 73% at 5 years [18]. The study period of this study was quite long (1970–1999), therefore, the radiation technique used in these patients vary and are most often not comparable to the current standard. The majority was treated with Cobalt sources and parallel fields. A more recent study [19] from the Netherlands described oncological outcome of 94 cT2 glottic cancer patients treated with radiotherapy between 2000 and 2012. Local control rate in this study was comparable to the Italian series, with a local control rate at 5 years of 70.5%. There are two main differences with the above mentioned series and the present series. The first is a possible bias regarding the tumor stage in relation to the treatment and fractionation. In our series we included only low volume cT2 tumors for hypofractionated radiotherapy. More bulky cT2 or those with impaired mobility are treated with conventional radiotherapy in our institute. According to our institutional guidelines, patients with a tumor with a volume of 2 cc or less should be treated with hypofractionated radiotherapy; nonetheless, 36% of the patients had a tumor volume above 2 cc. Most of these patients had tumors with volumes between 2 and 3 cc, but a subgroup analysis showed a non-significant trend towards loss of local control above 2 cc.

In addition, most of the cT1 tumors in our series are more bulky cT1a or cT1b, since most cT1a are preferably treated with CO_2_-laser surgery in our hospital. Therefore, most cT1 tumors are possibly the more unfavorable and for the cT2 tumors the more favorable. Besides, about a third of the patients had tumor involvement of the anterior commissure, a negative predictive factor (Bradley). Nonetheless, our results are comparable to published series in literature.

The second difference is the radiation technique used. The use of conventional fields or target volumes covering the conventional fields (i.e. whole larynx) with IMRT/VMAT in early-stage glottic cancer is still widespread and commonly used. A more conformal treatment of a margin based planning (vocal cord only) has led to a debate on safety versus benefit of this treatment [21]. Major concern in this respect was the risk of geographical misses due to delineation inaccuracy in combination with target motion. Target delineation is considered the weakest link in the chain of radiotherapy. Multimodal imaging could contribute to improved target delineation [24], but does not always lead to decreased inter observer variation [25]. Nonetheless, the use of diffusion weighted imaging in laryngeal cancer is promising and could possibly lead to more conformal delineations between observers since it provides sharper edges of tumorous tissue on imaging [17]. Target motion is a known problem in the head and neck area, especially in laryngeal cancer [26]. This intrafraction motion can be covered with patient-specific PTV margins based upon respiratory phase binned 4D-CT [27] or cine-MRI [28], or with (an)isotropic PTV margins of typically 5–7 mm. For fractionated radiotherapy, swallowing motion does not affect dose distribution, since swallowing occurs only rarely and takes a very short amount of time [26]. Therefore, most of the concerns raised in literature can be overcome with modern possibilities of radiotherapy like DWI and motion tracking. The present study shows that the clinical outcome is comparable to historical series. Nonetheless, this is not a randomized clinical trial meaning that firm conclusions cannot be drawn regarding the non-inferiority.

Mildly hypofractionated radiotherapy for cT1-2 tumors shows equivalent results in multiple series, including the present series. Nonetheless, results are significantly worse for cT2 tumors compared to cT1 tumors, so improvements for cT2 tumors are necessary in order to limit the need for salvage surgery. Patient selection for a hypofractionated schedule is a key factor. A clear volume cutoff of the GTV was not found in this study, but the clinically used 2 cc might be a useful cutoff. The finding that persistent smokers have had a higher risk of local failure is previously described [29]. Especially in larger tumors (cT2), this finding could suggest a negative role of hypoxia and radiation resistant subclones in T2 tumors. Future work need to focus on improvement of treatment results in this specific group of patients. The debate on more extreme hypofractionation towards stereotactic treatment of early-stage glottic larynx cancer is already ongoing. In the light of hypoxia as cause of treatment failure, stereotactic radiotherapy could be a solution to this, since hypoxia seem to play less of a role in the radiobiology of stereotactic radiotherapy [30]. A phase 1 dose-finding study [31] in 29 patients with Tis-T2 glottic larynx cancer already showed that a stereotactic radiotherapy regimen of 5 times 8.5 Gy was tolerable and resulted in good oncological outcomes. Median follow-up in the highest dose group was 25.7 months. Currently, a phase 2 study is enrolling patients to further study stereotactic radiotherapy for laryngeal cancer.

Toxicity in our series was very limited (4.3% grade III or higher late toxicity), and due to the retrospective character of the study, it was only possible to reliably report late toxicity. One patient underwent total laryngectomy due to organ dysfunction. In the series from Elicin et al., there were 3/761 patients who underwent total laryngectomy because of laryngeal dysfunction of whom one was treated with hypofractionated radiotherapy and two conventionally fractionated radiotherapy [22]. In the series from Hendriksma et al., late toxicity was reported in 5.3%, of which 2 patients developed laryngeal necrosis [19]. The laryngectomy-free survival at 5 years in that series was lower (74.7%) compared to the results of our series (90%). Although in the series from Hendriksma et al., only cT2N0 glottic carcinomas were included, which could explain the higher laryngectomy rate. The single vocal cord series on cT1a tumors reported 6.5% late grade III toxicity, with 4.5% laryngeal necrosis [15]. Although the 5-year laryngectomy-free survival was 98%. These numbers are slightly different due to the prospective design (higher reported toxicity) and the cT1a tumors (less laryngectomies).

### Limitations

Since this study is retrospectively performed, there is selection bias that may be of influence on the outcome of the study. Although we included consecutive patients, there may be some selection based on none definable factors. In addition, toxicity was not scored in a standardized manner, therefore, it was chosen to only report objective toxicity like tube feeding, hypothyroidism, tracheostomy and laryngectomy. In addition, voice quality (e.g. by scoring the voice handicap index) is not registered in all patients. Lastly, the statistics were hampered by limited number of patients included in the study. Multivariable analysis was not possible, due to the low number of recurrences in our cohort.

## Conclusion

Vocal cord-only hypofractionated radiotherapy appears to be oncologically safe in early-stage glottic laryngeal squamous cell carcinoma. The use of modern, image-guided radiotherapy led to comparable results as historical series on entire larynx and single cord irradiation with very limited late toxicity. Oncological outcomes in patients with cT1 and T2 glottic laryngeal carcinoma are good, but in patients with cT2 tumors, besides the proposed 2 cc volume cutoff, better patient selection criteria should be developed.

## Data Availability

The datasets generated during and/or analysed during the current study are available from the corresponding author on reasonable request.
